# New discovery of two seismite horizons challenges the Ries–Steinheim double-impact theory

**DOI:** 10.1038/s41598-020-79032-4

**Published:** 2020-12-17

**Authors:** Elmar Buchner, Volker J. Sach, Martin Schmieder

**Affiliations:** 1 HNU - Neu-Ulm University of Applied Sciences, Wileystraße 1, 89231 Neu-Ulm, Germany; 2 Meteorkrater-Museum Steinheim, 89555 Steinheim am Albuch, Germany; 3 Fokus Natur, In der Talwiese 2, 72488 Sigmaringen, Germany; 4 Lunar and Planetary Institute - USRA, Houston, TX 77058 USA

**Keywords:** Natural hazards, Planetary science, Solid Earth sciences

## Abstract

The Nördlinger Ries and the Steinheim Basin are widely perceived as a Middle Miocene impact crater doublet. We discovered two independent earthquake-produced seismite horizons in North Alpine Foreland Basin deposits potentially related to both impacts. The older seismite horizon, demonstrated to be associated with the Ries impact, is overlain by distal impact ejecta in situ, forming a unique continental seismite-ejecta couplet within a distance of up to 180 km from the crater. The younger seismite unit, also produced by a major palaeo-earthquake, comprises clastic dikes that cut through the Ries seismite-ejecta couplet. The clastic dikes may have formed in response to the Steinheim impact, some kyr after the Ries impact, in line with paleontologic results that indicate a time gap of about 0.5 Myr between the Ries and Steinheim events. This interpretation suggests the Ries and Steinheim impacts represent two temporally separate events in Southern Germany that, thus, witnessed a double disaster in the Middle Miocene. The magnitude–distance relationship of seismite formation during large earthquakes suggests the seismic and destructive potential of impact-induced earthquakes may be underestimated.

## Introduction

**Figure 1:**
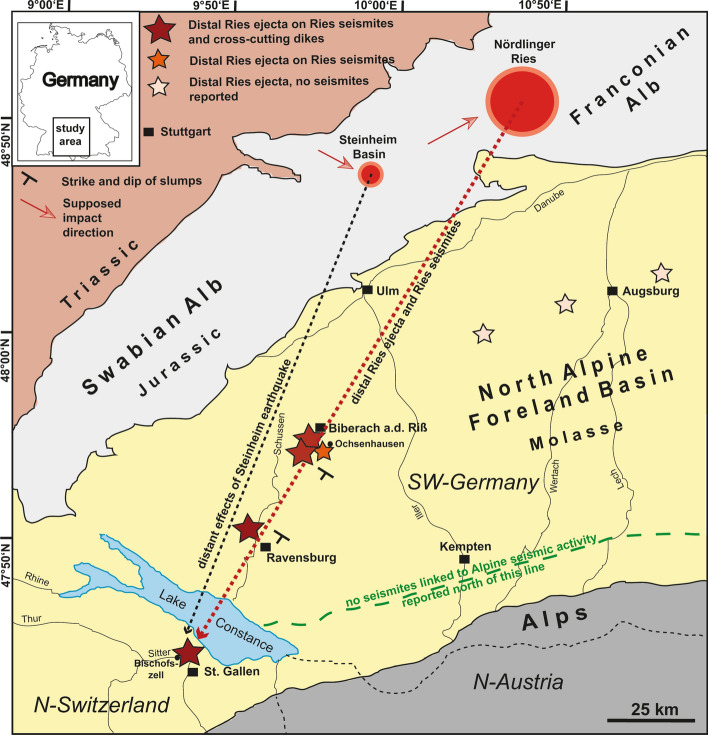
Geographic and geologic situation in the study area in southern Germany and northern Switzerland and Austria. Outcrops with Ries seismites overlain by the distal Ries ejecta layer (DREL), in turn cross-cut by clastic dikes presumably linked to the Steinheim impact^15^, are situated within a distance of 80 to 180 km from the centres of the two impact structures. Supposed different impact directions (orange arrows) of Ries and Steinheim asteroids are taken from the literature^1,8^ and are discussed in detail within these studies.

The ~ 24 km-diameter Nördlinger Ries^1–4^ and the ~ 4 km-diameter Steinheim Basin^1,5–8^ impact structures in southern Germany (Fig. 1) count among the best-preserved impact structures on Earth. Groundbreaking insights into impact crater and ejecta formation and shock metamorphic processes were gained from the study of these two structures^1–13^. The complex Ries crater is characterized by a well-preserved, double-layer ejecta blanket^4^ that comprises lithic impact breccia derived mainly from weakly shocked Jurassic to Triassic sedimentary target rocks, as well as by the overlying suevite that is mostly composed of variably shocked and partly impact-melted material derived from the crystalline crater basement. Impact melt occurs in various forms, including tektites found in the Central European tektite strewn field^9,14^. A conspicuous marker bed is the coarse-grained distal Ries ejecta layer (henceforth DREL; locally known as the ‘Brockhorizont’, ‘Blockhorizont’, and ‘Reuter Blocks’)^10,11,15–18^, a locally reworked horizon of sand, pebbles, cobbles, and boulders of predominantly Upper Jurassic limestone. The DREL components were ballistically transported over distances up to 180 km, deposited and preserved in the siliciclastic sediments of the North Alpine Foreland Basin.

The ~ 4 km-diameter Steinheim Basin, ~ 40 km SW of the centre of the Ries crater, is a complex impact crater with a prominent central uplift set in a sequence of Triassic and Jurassic sedimentary rocks^5–8^. The Steinheim Basin is well known for its shatter cones of outstanding shape and quality^5,7,8^. Impact breccias are known from numerous drillings into the Steinheim Basin^5–7^ containig variable amounts of clasts of Jurassic limestones, marls, mudstones, and sandstones. The morphological crater rim exhibits inclined and brecciated blocks and clods of Upper Jurassic (Kimmeridgian–Tithonian) marine limestones^5,6^. Although isotopic dating failed to yield a geologically meaningful age, the Steinheim Basin is thought to have formed simultaneously with the Nördlinger Ries crater^1–3,8^ at 14.808 ± 0.038 Ma^12,13^. The general notion is that the crater pair was formed by the impact of a binary asteroid of ~ 1 km and ~ 100–150 m in diameter, respectively^1,2^. In other studies, however, it was pointed out that the simultaneous formation of the two impact structures is still uncertain^5,7,8,19^.

A hallmark of large impact events are layers of ejected and partially melted target rock material in the Earth’s sedimentary record, including tektites, impact spherules, and shocked mineral grains^9,14,20–22^. While a number of such desposits are known on Earth^14–16^, distal impact ejecta that contain larger target rock fragments ballistically transported over more than 100 km (or even some hundreds of kilometres) are sparsely reported in the literature. Most reports of distal impact ejecta stem from sedimentary successions encompassing the Cretaceous-Paleogene (K–Pg) boundary in the wider surroundings of the 180 km-diameter Chicxulub crater, linked with the end-Cretaceous mass extinction^23–26^. Distal air-fall ejecta penetrating sedimentary deposits at the K–Pg boundary occur in the Hell Creek Formation (North Dakota, USA) about 3,000 km from Chicxulub^20^. The ejecta horizon of the Ediacaran ~ 90 km-diameter Acraman impact structure in South Australia contains shocked mineral grains and shatter cones in clasts at distances as far as > 500 km from the source crater^21^.

Various outcrops that include the DREL^10,11,15–18^ are known from the North Alpine Foreland Basin^27,28^ in southern Germany and northern Switzerland within a maximum distance of 180 km from the Nördlinger Ries^2,10,11^. Most of these ejecta components are Upper Jurassic limestone derived from the upper portion of the Ries target rock, some of them with shatter cones^11,16^ confirming their origin as impact ejecta and suggesting shock pressures of at least ~ 2 GPa^29^.

Another effect of large asteroid impacts are intense earthquakes^2,3,20,30–34^. The giant Chicxulub impact is thought to have generated a seismic pulse roughly equivalent to a magnitude M_W_ (moment magnitude scale) 10–11.5 earthquake^20^. According to equations provided in a web-based computer program to calculate the regional environmental consequences of an asteroid impact on Earth^30^, the impact that formed the 24 km-diameter Ries crater likely caused a magnitude M_W_ ~ 8.5 earthquake^30^. For the much smaller Steinheim impact event, the calculated earthquake magnitude is approximately M_W_ 6.6^30^ (for estimates of the moment scale magnitudes M_W_ for the Ries and the Steinheim impact events see Supplementary Table 1).

Impact-triggered earthquakes produce seismites in extensive volumes of sediment that are in many ways similar to seismites generated by tectonically-induced earthquakes. Cosmic impacts can produce clastic dikes^32^ proximal to the impact structures^15,31–33,36^ and may also cause soft-sediment deformation by liquefaction at greater distances from ground zero^15,17,20–23,31–34,36^; however, the style of deformation is in part governed by the nature of the near-surface sediments (e.g., diagenesis/cemenation, grain size, water saturation). Although the Ries and Steinheim impact events would have triggered significant earthquakes, there is only emerging evidence for palaeo-earthquakes in the surroundings of the two impact structures in the form of seismites. Recently, a clastic dike was discovered in sandy deposits of the North Alpine Foreland Basin and interpreted as an impact-related seismite^15^. That dike cuts through the DREL and might, thus, represent a long-distance effect of the Steinheim impact event that appears to postdate the Ries impact by several kyr^15^. We here present additional evidence for two separate seismite horizons exposed at several localities within the North Alpine Foreland Basin in southern Germany and northern Switzerland. Both seismite occurrences are consistent with at least two strong, independent palaeo-earthquakes.

## Results and discussion

### Ries-related seismite

**Figure 2:**
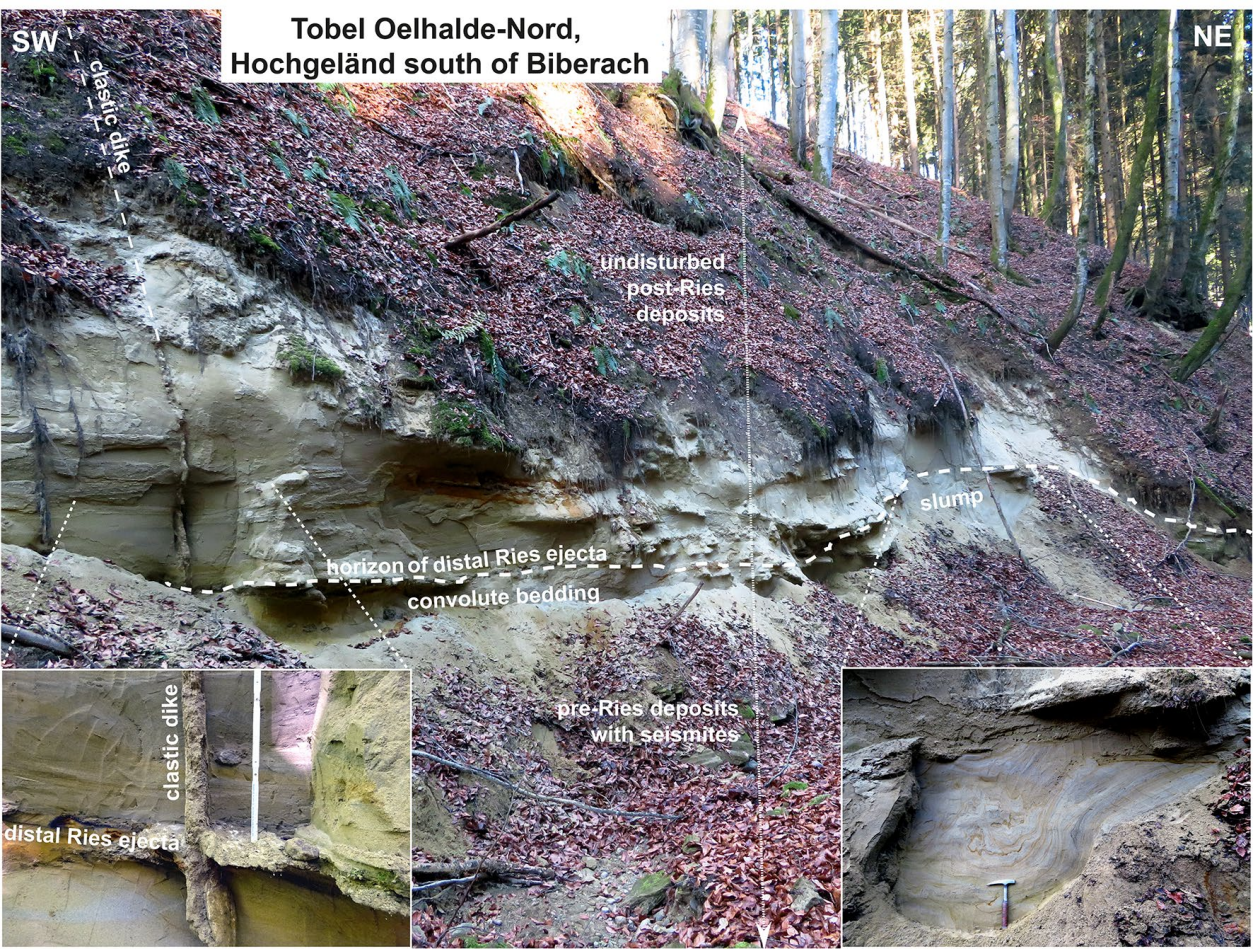
Bedding within sandy sediments in the Tobel Oelhalde-Nord south of Biberach (Tobel is the local term for a small ravine in southern Germany), approximately 100 km SSW of the Ries crater rim. Slumped deposits of Upper Freshwater Molasse with soft-sediment deformation structures of pre-Ries age are overlain by the in-situ DREL and essentially undisturbed deposits of post-Ries age. The DREL traces the relief of the pre-Ries land surface. A clastic dike presumably linked to the Steinheim impact^22^ that postdates the Ries impact crosscuts the entire succession. Photographs taken by V.J.S.

**Figure 3:**
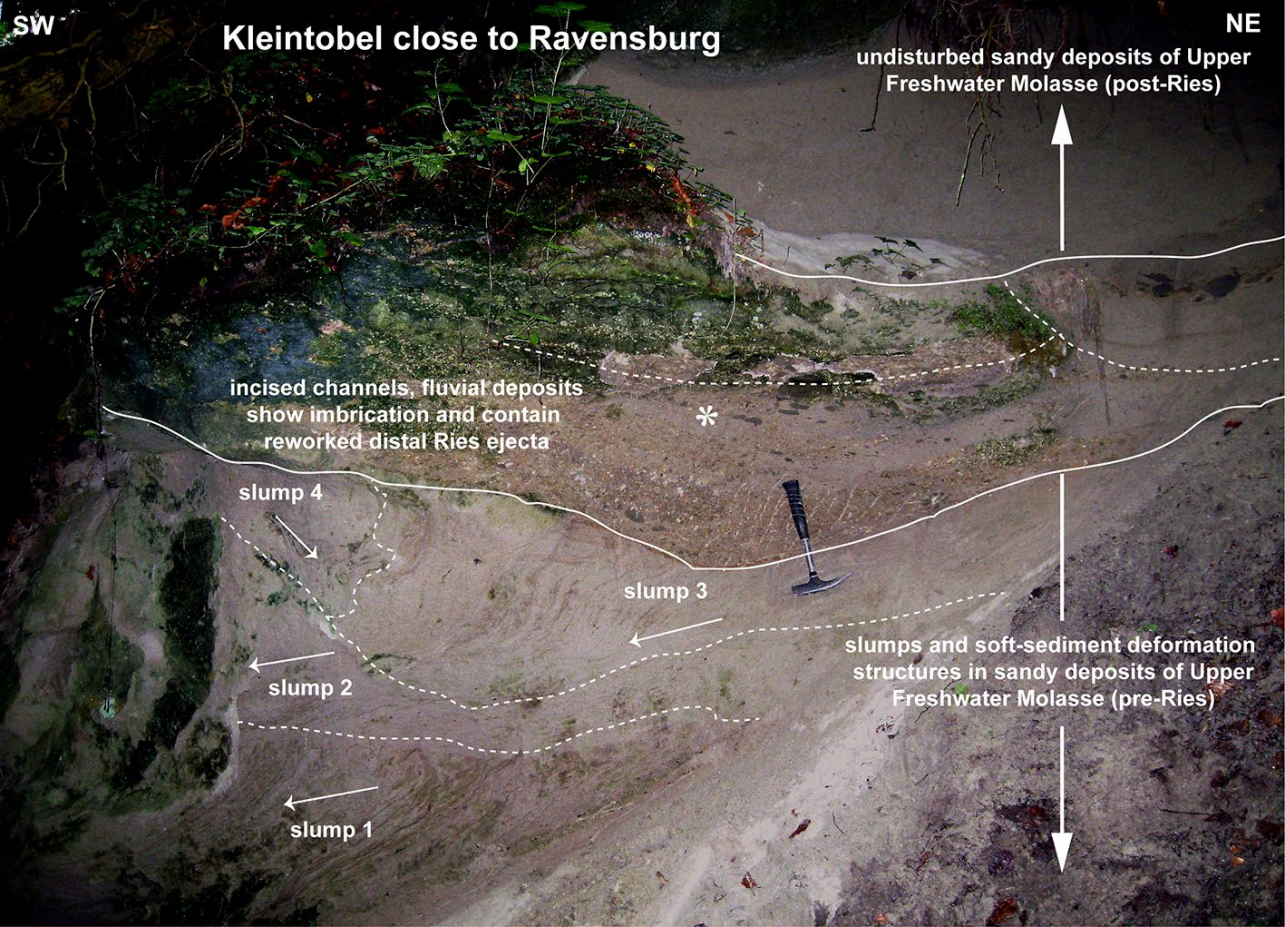
Bedding conditions in the Kleintobel close to Ravensburg, approximately 130 km SSW of the Ries crater rim. Channel-fills with reworked distal Ries ejecta are incised into slumped deposits of Upper Freshwater Molasse with soft-sediment deformation structures of pre-Ries age and show distinct imbrication (asterisk). Arrows show flow direction of slumps which generally tend towards the SW. Reworked distal Ries ejecta is overlain by undisturbed post-Ries deposits. Photograph taken in Kleintobel south of Biberach by V.J.S.

**Figure 4:**
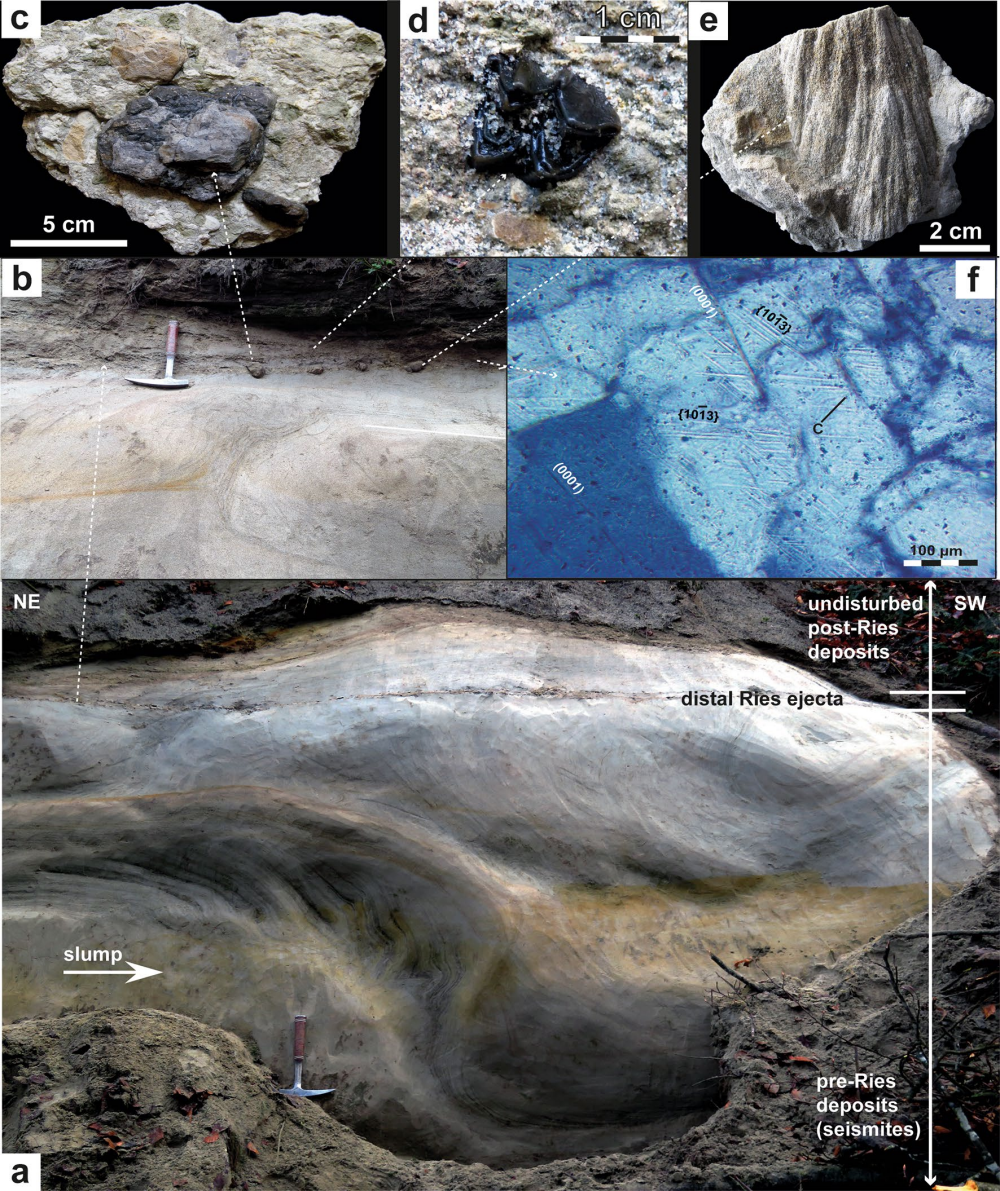
(**a**) Bedding conditions in the Kleintobel close to Ravensburg approxiamtely 140 km SSW of the Ries crater rim. Slumped deposits of Upper Freshwater Molasse overlain by a layer of distal Ries ejecta in situ and undisturbed deposits of post-Ries age (see hammer for scale). (**b**) Cobbles of distal Ries ejecta in situ that impacted into slumped deposits of Upper Freshwater Molasse. Note the about 5 cm deep impact depression under one of the DREL clasts (left). (**c**) Two clasts of distal Ries ejecta, one of Upper Jurassic limestone (light) and the other of Lower Jurassic claystone (dark), both connected by secondary carbonate cements. (**d**) Molar tooth of a Middle Miocene deer (*Heteroprox sp.*). (**e**) Shatter cone in an ejected cobble of Upper Jurassic limestone. (**f**) Shocked quartz grain with at least three sets of planar fractures and planar deformation features and their crystallographic orientation from the sandy portion of the Ries ejecta horizon at this locality. Photographs (**a**–**e**) taken by V.J.S. and (f) by E.B.

**Figure 5:**
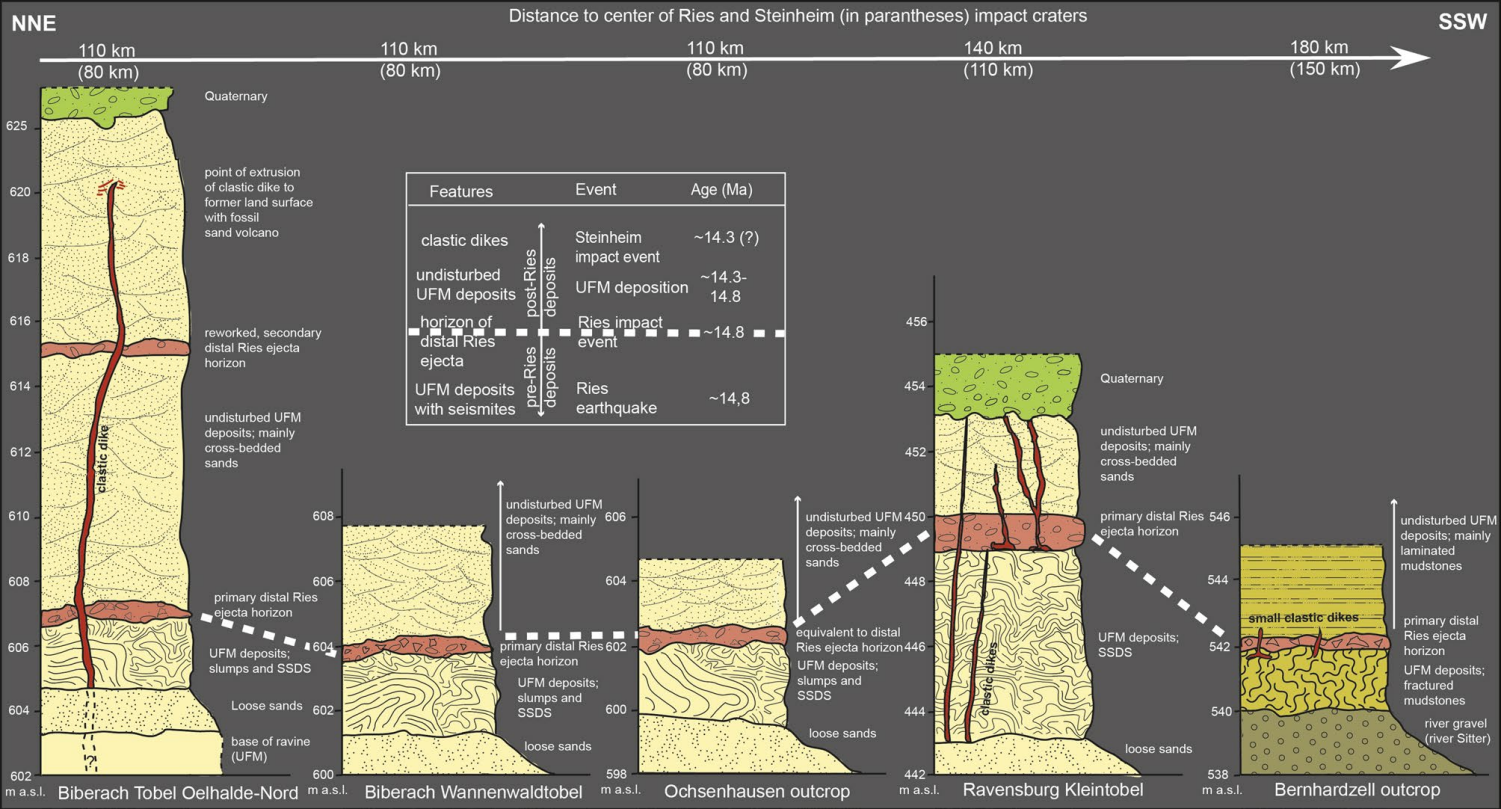
Schematic cross sections of the outcrops containing Ries-related seismites capped by primary and reworked distal Ries ejecta and undisturbed deposits of Upper Freshwater Molasse. Clastic dikes, presumably linked to the Steinheim impact^15^, cut through the seismites and Ries ejecta in three different outcrops; UFM: Upper Freshwater Molasse; SSDS: soft-sediment deformation structures; Tobel is the local term for a small ravine in southern Germany).

We discovered sedimentary successions with distinct soft-sediment deformation structures in a temporary construction site near Ochsenhausen^15^, in three ravines (locally called ‘Tobel’ in southern Germany) at the ‘Tobel Oelhalde-Nord’ and ‘Wannenwaldtobel’ close to Biberach an der Riß (Figs. 1, 2, Supplementary Fig. 1), and at the ‘Kleintobel’ near Ravensburg (Fig. 3, Supplementary Fig. 2). The discovery of one large clastic dike from the ‘Tobel Oelhalde-Nord’ was described by our group in an earlier study^15^. The soft-sediment deformation structures include metre-sized slumps (Figs. 2, 3, 4), all with NW–SE-striking slump axes (Figs. 1, 2), convolute bedding, ball-and-pillow and flame structures, and clastic dikes. The dip of the slumps and the strike of the slump axes (Fig. 1) are consistent with a seismic source in the Ries–Steinheim region and are, therefore, unrelated to a source region in the Alps and nearby intracontinental volcanic fields that were active during the Miocene. Such soft-sediment deformation features in continental deposits are typical of seismites caused by large earthquakes^15,32,37^. As an analog example, soft sediment deformation (slumps) with preferred orientations of slump-fold axes perpendicular to the probable epicentre (the Manicouagan impact event in eastern Canada) were reported in latest Triassic (Rhaetian) deposits in central Britain^34^. The DREL that caps the seismite unit (Figs. 2, 4, 5) provides compelling evidence that the Ries impact was the source for this seismic event, causing soft-sediment deformation within a radial distance of ~ 100 to 180 km from the impact site. The restricted occurrence of the seismite horizon within the study area may reflect variable properties of the near-surface Molasse sediments within the North Alpine Foreland Basin (as opposed to an area-wide distribution in the surroundings of the Ries and Steinheim craters) and is discussed in detail in chapter ‘Distribution of seismites’ in the Supplementary Material.

### Distal Ries ejecta

The DREL^10,11,15–18^ was described from several outcrops in the Middle Miocene Upper Freshwater Molasse of the North Alpine Foreland Basin in Bavaria^18,38,39^ (SE Germany), Baden-Württemberg^11,15,16^ (SW Germany), and NE Switzerland^17^. During field work, we found additional outcrops of distal Ries ejecta in three ravines south of Biberach an der Riß and west of Ravensburg, recpectively. In addition to the larger cobbles and bolders at the base of the cm- to dm-thick primary ejecta horizon, the ejecta layer also consists of sand and small pebbles mainly made up of grains of limestone, quartz, and feldspar^15^. These finer-grained deposits locally show a distinct fining-upward trend. Quartz grains in the ejecta horizon are often very angular and show a weak to moderate shock overprint (e.g., indistinct planar deformation features in one or two directions) in agreement with pressures at the lower end of the shock metamorphic regime (mostly < 5 GPa). Only a small proportion of quartz grains in the distal Ries ejecta horizon of the study area show a higher degree of shock-metamorphic overprint in the form of planar deformation features in up to six optically visible directions (Fig. 4f, Supplemetary Fig. 5). These highly shocked quartz grains were probably derived from the crystalline basement and, hence, from deeper parts of the Ries target (at least ~ 600 m below the former land surface). At all outcrop sites analyzed in this study, distal Ries ejecta overlie a seismite unit, thereby forming a distinct seismite-ejecta couplet. The ejecta horizon occurs either as a primary, in situ (Fig. 2), or secondary (fluvially reworked; Fig. 3) layer of ejecta^16^. At the Tobel Oelhalde-Nord (Biberach; Fig. 2), Wannenwaldtobel, and Kleintobel (Ravensburg; Figs. 3, 4), angular clasts (Supplementary Fig. 3) of Upper Jurassic limestone locally produced small dents (Fig. 4b) caused by the impact of Ries-ejected pebbles, cobbles, and boulders (Fig. 4c) into the soft sediment after ballistic air-travel over > 100 km^10,15,16,18^. Some of the clasts (mainly of Upper Jurassic limestones) contain shatter-cones (Fig. 4e). These observations suggest the seismite in the underlying pre-Ries deposits is genetically related to the Ries impact. The exposures of the seismite-ejecta couplet are situated within a distance of ~ 100 km (Ochsenhausen), ~ 110 km (Biberach), and ~ 140 km (Ravensburg) from the centre of the Ries crater, respectively (Fig. 1). The most distant known occurrence of coarse-grained Ries ejecta occurs ~ 180 km SSW of the Ries crater, in an outcrop near Berhardzell in NE Switzerland (Fig. 1) from which shocked quartz grains were reported (pers.comm. Carl Alwmark). In this study, we present new evidence for shocked quartz grains with up to four sets of planar deformation features in loose sands constituting Ries ejecta exposed in the Tobel Oelhalde-Nord (Biberach; Fig. 4f, Supplementary Fig. 3), and with up to six sets of planar deformation features in Ries ejecta from the Kleintobel (Ravensburg, Supplementary Fig. 5). The Upper Freshwater Molasse deposits that overlie (i.e., postdate) the DREL are typically cross-bedded or horizontally layered and generally appear undisturbed and unaffected by dewatering processes.

### Clastic dikes

In addition to the seismite capped by distal Ries ejecta, we discovered outcrop-scale clastic dikes first described along the flanks of the Tobel Oelhalde-Nord near Biberach^15^ and at the Kleintobel near Ravensburg (this study). These clastic dikes are earthquake-produced structures^15,32^ that crosscut the Ries-related seismite, ejecta (the DREL), and undisturbed post-Ries deposits and, hence, clearly postdate the Ries impact event and earthquake. A horizon of distal Ries ejecta associated with smaller clastic dikes is also known from Bernhardzell, Switzerland^17^. Those dikes also seem to postdate the Ries impact and, overall, the local facies and structural situation resemble those at Biberach and Ravensburg^15^. The genetic relationship between the seismite-hosting deposits and the Ries impact is evidenced by the primary (Supplementary Fig. 4) DREL in situ sitting right on top of these deposits. The large clastic dike that cross-cuts both the Ries seismite and ejecta near Biberach was recently tentatively linked to the somewhat younger Steinheim impact^15^. Notably, this scenario—suggesting two spatially and temporally separate impacts—challenges the widely accepted binary asteroid hypothesis for the Ries–Steinheim event^1–3,8^.

### The Ries and Steinheim craters: not the binary asteroid impact it seems?

**Figure 6:**
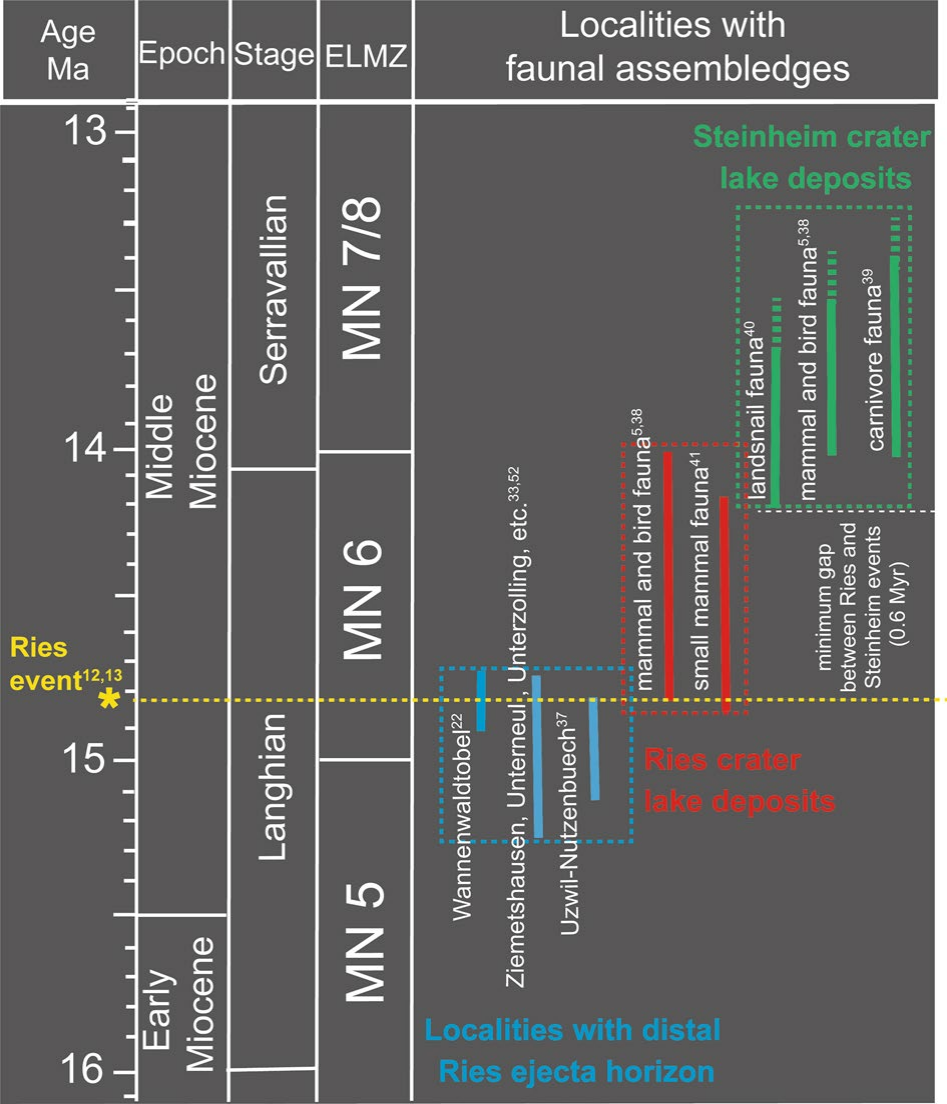
Faunal assemblages (European Land Mammal Zones, ELMZ) that occur within the Ries and Steinheim crater lake deposits and in context with the distal Ries ejecta horizon. Beside mammals, the ELMZ also comprise the typical floral and faunal (e.g., birds, snails) assemblage for each zone.

The distinct SW–NE alignment of the Steinheim Basin, the Nördlinger Ries impact structure, and the Central European tektite strewn field seemingly supports the general notion that both impact structures represent an impact crater doublet formed by an incoming pair of asteroids entering the Earth’s gravitational field from the SW^1,8^. While a precise and accurate ^40^Ar/^39^Ar age has been established for the Ries impact (14.808 ± 0.038 Ma^12,13^), isotopic dating has, thus far, failed to yield a geologically meaningful age for the Steinheim impact. Several studies pointed out that the simultaneous formation theory for the two impact structures is, in fact, not evidenced by palaeontologic and structural geologic constraints^5,38,40^ (and references therein). From a biostratigraphic point of view, the Steinheim impact could postdate the Ries impact by as much as 1 Myr^5^. The oldest lake deposits inside the Ries crater contain a fossil fauna that belongs to the transition of the mammal zones MN 5 to MN 6 (Langhian stage of the Miocene), whereas fossils in the basal lake deposits of the Steinheim Basin correspond to the transition of mammal zones MN 6 to MN 7^15,16,38,40,41^ (Serravallian stage of the Miocene). These biostratigraphic ages indicate a time gap of at least ~ 0.6 Myr^5,15,38,40^ (Fig. 6) between the formation of both craters, which is in obvious conflict with the double-impact scenario^5,8,15^. A NW–SE-trending impact direction proposed for the Steinheim Basin^8^, as well as possibly differing impactor traces at both impact sites (i.e., a possible pallasite as the Steinheim meteorite^6,8^ vs. a missing or achondritic impactor signature for the Ries^2,3,6,8^) are no firm evidence against the double impact scenario, but are more consistent with two separate impact events.

Both the Nördlinger Ries and the (possibly) slightly younger Steinheim impacts would have imparted significant energy into the sedimentary target, causing at least regional-scale disturbances. Although seismites linked to Alpine seismotectonic activity have been reported in the literature^15^ (references and discussion therein), no such seismites are known north of the line Lake Constance – Oberstaufen – Immenstadt^15^ (see Fig. 1, green line). However, as described in this study, a laterally extensive seismite occurs in sandy deposits of the Upper Freshwater Molasse of pre-Ries age several tens of kilometres north of that line (near Biberach, Ochsenhausen, and Ravensburg) and is capped by a primary horizon of distal Ries ejecta in situ and undisturbed younger deposits. This suggests the seismite is the product of a Ries impact-induced giant earthquake. At Biberach^15^, Ravensburg, and Bernhardzell^17^, clastic dikes cut through the Ries-related seismite-ejecta couplet and portions of the overlaying Upper Freshwater Molasse. This provides tangible evidence for a second, high-magnitude earthquake in the region that had previously been affected by the ‘Ries earthquake’. The Biberach clastic dike exposed at the Tobel Oelhalde-Nord reached the former land surface forming an extrusive fossil sand volcano^15,42^. Based on the age constraints for the dike-hosting sediments^15,16,39^ the dike is the product of a seismic event that occurred between ~ 14.81 Ma (Ries impact^12,13^) and approximately 14.3 Ma (terminal sedimentation of the ‘Fluviatile Untere Serie unit^15,39^). In contrast to the precise isotopic age for the Ries^12,13^, the latter age is not very well constrained and may be associated with an error of a few kyr^15^. A seismo-tectonic (alpine tectonism) or volcano-seismic event (within the Paleogene to Quaternary European Volcanic Province) was recently discussed^15^ as a potential source for the younger earthquake some ~ 0.5 Myr after the Ries impact. However, considering their distant geographical position and rather low seismic potential^15^, none of these earthquake centres can convincingly explain the formation of the post-Ries clastic dikes^15^.

The dimensions of sandstone dikes significantly decrease towards the South, from the giant Biberach clastic dike in the North and the dikes near Ravensburg to the dm-long clastic dikes of Bernhardzell in Switzerland. These localities are situated at 80 km, 110 km, and 150 km south of the Steinheim crater, respectively. Dike dimensions are a function of host rock properties and seismic energy^15,32,33^. Taking the comparable rock properties and the significantly different dimensions of the clastic dikes at the three localities into account, the seismo-tectonic epicentre was likely located north of the Biberach area. This renders a seismic source in the northern Alps that could be responsible for the formation of the dikes in the study area less likely. The only volcanically active region in the Middle Miocene north of the study area is the ~ 18 to 14 Ma phreatomagmatic Urach-Kirchheim volcanic field consisting of more than 350 tuffaceous and olivine-melilititic maar-diatreme complexes^15^. Due to the relatively low seismic efficiency of phreatomagmatic volcanism, intense and long-distance seismic effects of that volcanism are also unlikely^15^ (see discussion and references therein). This suggests the Steinheim impact, which seemingly has the right position and approximate age, may have been the trigger of the post-Ries seismic event^15^.

Supporting arguments for a major post-Ries seismic event come from sediments of the lake inside the Ries crater itself. A ~ 314 m-thick sequence of crater lake deposits was drilled in the scientific drilling project 1973. This sediment sequence, deposited in a lake that lasted for ~ 1 Myr^43,44^, contains olistoliths and sediments with intense slumping and convolute bedding^44^. Somewhat surprisingly, the slumped deposits do not occur at the basis of the lake deposits, which would have been favored by the steep relief of the newly formed, precipitous impact crater; but soft-sediment deformation appears to be dominant in the middle of the sedimentary succession. The slumps and convolute bedding within the Ries crater lake could well represent a long-distance effect of a strong earthquake some hundred kyr after the Ries impact, potentially triggered by the Steinheim impact only some 40 km SW of the Ries crater.

The two major paleoseismic events recorded at various sites across the North Alpine Foreland Basin seem to have occurred close in time in the Miocene, yet during markedly different climatic and paleoenvironmental conditions. Soft-sediment deformation caused by the Ries earthquake at ~ 14.81 Ma occurred when the climate was warm and humid^45–49^ (during or slightly after the Miocene Climate Optimum at 14.9 Ma^46–48^) and the palaeo-groundwater level reached the former land surface. While the Ries-triggered earthquake caused extensive stirring of water-saturated sediments, the earthquake presumably induced by the Steinheim impact seemingly did not cause any widespread soft-sediment deformation, but generated clastic dikes. This suggests a rather dry state of the sedimentary bedrock, with a deeper palaeo-groundwater level locally above water-logged clay and silt horizons. An episode of significant climate change during the Middle Miocene in Central Europe was recently dated at ~ 14.48 to 14.13 Ma^46–48^ through the analysis of palaeosoils in the North Alpine Foreland Basin. That change in climate led to a stronger seasonality and less humid conditions in Central Europe^46–48^. Assuming the Steinheim impact and the Biberach clastic dike are genetically linked, the age for the Steinheim impact would most likely fall between ~ 14.8 and ~ 14.1 Ma. Taking the biostratigraphic, sedimentologic, and climatologic findings into account, the suggested best-fit impact age for Steinheim is approximately 14.3 Ma. This age fits well with the time frame of the terminal sedimentation of Fluviatile Untere Serie at 14.3 Ma^39^ and the initial phase of Mid-Miocene cooling at 14.43 Ma^46–48^. The time gap of approximately 0.5 Myr also fits the purported age difference between the crater lake deposits at both impact structures, as well as the post-Ries timing of active slumping within the Ries crater lake sediments. All these arguments, combined with the lack of an effective seismic source for a high-magnitude earthquake postdating the Ries event, lead us to conclude that the Ries and Steinheim impact structures might be the result of two temporally separate impact events in southern Germany, occurring ~ 40 km and ~ 0.5 Myr (and up to 1 Myr?) apart.

In the past decade, many of the seemingly well-established terrestrial impact crater doublets and chains were discredited despite the seemingly low calculated likelihood of two separate impacts spatially close to one another^50–52^. ^40^Ar/^39^Ar dating results for several impact structures^51–54^ contradict the hypothesis that planet Earth experienced the formation of a giant ‘impact crater chain’ during a major Late Triassic multiple impact event^50^. Recent work, moreover, revealed that apparent crater pairs, for instance the partly overlapping East and West Clearwater Lake impact structures (Québéc, Canada)^51^ or the two Suvasvesi impact structures (Finland)^52^, are not the crater doublets they seem. To date, the only terrestrial crater pair that survived closer inspection is the Lockne–Målingen pair in Middle Sweden^36^, which was produced during an active period of Mid- to Late Ordovician asteroid bombardment of the Earth^53,54^.

Assuming two spatially and temporally separate impact events, the occurrence of the distinct and well-preserved Ries-related seismite topped by primary distal ejecta near Biberach, Ravensburg, and Bernhardzell is explained as follows: 1. Thick, fine-grained, and homogenous sandy deposits intercalated with clays^15^ promoted water-saturation within the Upper Freshwater Molasse in the study area, facilitating dewatering processes and soft-sediment deformation^15^ triggered by the Ries impact. 2. Distal Ries ejecta blanketed the Ries seismite, was locally preserved in situ, and presently crops out in ravines and a river bank. 3. As an additional feature, clastic dikes^15,32,55^ cutting through the Ries-related seismite-ejecta unit appear to have been caused by a second high-magnitude earthquake presumably linked to the Steinheim impact some kyr after the Ries impact event^5,15^. The occurrences of the seismite near Biberach, Ochsenhausen, Ravensburg, and Bernhardzell are the first reported deposits in which evidence for earthquake-induced soft-sediment deformation structures caused by the Ries impact has been documented. To our knowledge, this is also the first known occurrence of a primary continental seismite-ejecta couplet exposed in situ.

### Magnitudes of impact-earthquakes

The magnitude of earthquakes induced by meteorite impacts is still somewhat uncertain, and the seismic efficiency (i.e., the portion of the impactor’s kinetic energy transformed into seismic energy) is only constrained within two orders of magnitude (for the theoretical background and calculations see “Methods” section)^15,30^. Accordingly, taking into account global-scale seismic effects linked (tentatively) with terrestrial impacts^2,3,14,20,22,23,30,31,33,56^, calculated magnitudes may, in some cases, be too conservative^15^. Applying widely used equations, the magnitude of the ‘Chicxulub earthquake’ was probably approximately M_W_ 10–11.5^20^ (and references therein). Endogenic (tectonic) earthquakes may not reach such an extraordinary magnitude^57,58^, and the strongest earthquakes ever recorded correspond to a magnitude M_W_ 9.2 to 9.5 (e.g., the Alaska earthquake (USA) or the Great Chilean (Valdivia) earthquake^59–61^_;_ see Supplementary Table 1).

An earthquake of a moment magnitude of M_W_ 6.5 or higher is required for the formation of seismites^15,32^. The systematic relation between specific styles of crustal deformation (e.g., clastic dikes and soft-sediment deformation) and radial distance from the seismic source depending on the earthquake magnitude was studied for many regions on Earth^55,59,60^ and takes into account the decrease of energy of seismic waves with time and rock volume traveled. Liquefaction and the concomitant formation of seismites caused by impact-induced earthquakes is preserved in the sedimentary record at a number of localities worldwide and summarized in a comprehensive database^15,17,20,22,23,25,31,33,34,62,63^. However, the earthquake magnitude–distance relationship for liquefaction effects is currently still underexplored and needs to be evaluated from the perspective of geologically younger major earthquakes.

For the impact that formed the 24 km-diameter Ries crater in southern Germany (impact energy ~ 5 × 10^23^ J; equivalent to ~ 120,000 megatons of TNT), an earthquake of moment magnitude M_W_ ~ 8.5 was calculated^30^ (Supplementary Table 1). The most distal exposures of a seismite in the form of soft-sediment deformation structures and clastic dikes caused by the Ries impact-induced earthquake occur within a distance of at least 180 km from the centre of the crater (Bernhardzell, Switzerland). According to the mapping of distal ground failure effects caused by large earthquakes up to M_W_ ~ 7.8 (M_L_ 7.5), clastic dikes and soft-sediment deformation structures may occur at a distance of ~ 70 to 130 km from the epicentre of major earthquakes^55–60^. Even the giant 1964 Alaska earthquake that had a magnitude of M_W_ 9.2^61^ caused significant ground failure only within a radius of 130 km^59,60^. On the other hand, earthquakes that caused liquefaction of sediments within a radial distance of more than 150 km all had magnitudes of M_W_ ~ 8.5 or higher^59,60^. A moment magnitude of M_W_ ~ 8.5 for the eroded, ~ 10 km-diameter Upheaval Dome impact structure^31,62^ in Utah, USA was proposed on the basis of the earthquake magnitude-distance relationship for synsedimentary deformation in Jurassic rocks in the wider surroundings of the impact site^31^. Taking these arguments into account, a magnitude in the range of M_W_ ~ 8.5 (and perhaps even higher) for the ‘Ries earthquake’, producing seismites within a 180 km radius, appears geologically plausible. Based on the comparison with distal ground effects of historical earthquakes^59,60^, a local magnitude in the range of the 1964 Alaska earthquake (M_W_ 9.2^61^) might be the best endogenic analog for the Miocene Ries earthquake and its distant effects.

The nearby Steinheim impact event (impact energy ~ 2.3 × 10^18^ J; equivalent to ~ 550 megatons of TNT) formed a much smaller, complex impact crater about 4 km in diameter. The magnitude of the Steinheim earthquake^15^ was estimated at about M_W_ ~ 6.6^30^ (Supplementary Table 1). The most distal seismites in the form of soft-sediment deformation and clastic dikes presumably linked with the Steinheim impact earthquake occur within a radial distance of at least 150 km from the source crater. While ground failure due to earthquakes of M_W_ ≥ 7.8 may occur within a radial distance of 100 km or more, the outer limit for the occurrence of seismogenic clastic dikes dramatically decreases for earthquakes of M_W_ < 7.8^59,60^. The most distal ground effects of an earthquake with M_W_ 7.1 (Supplementary Table 1), for instance, reach radial distances of only ~ 23 km from the epicentre^59,60^. The formation of clastic dikes at a radial distance of 150 km, therefore, requires a palaeo-earthquake of the magnitude M_W_ ~ 8.5 or higher. From this point of view, we speculate whether the magnitude of the postulated ‘Steinheim earthquake’, assuming a genetic link, may have been (significantly) higher than M_W_ 6.6^15,30^. A remaining caveat is that precise and accurate calculations of the seismic intensity of impact events are not straightforward, because the knowledge about the near-surface propagation of seismic waves following impact events is rather limited^15,30^ and the seismic efficiency factor (determined within an uncertainty of three orders of magnitude^15,30^) is not well constrained.

### Environmental effects of the Ries and Steinheim events

**Figure 7:**
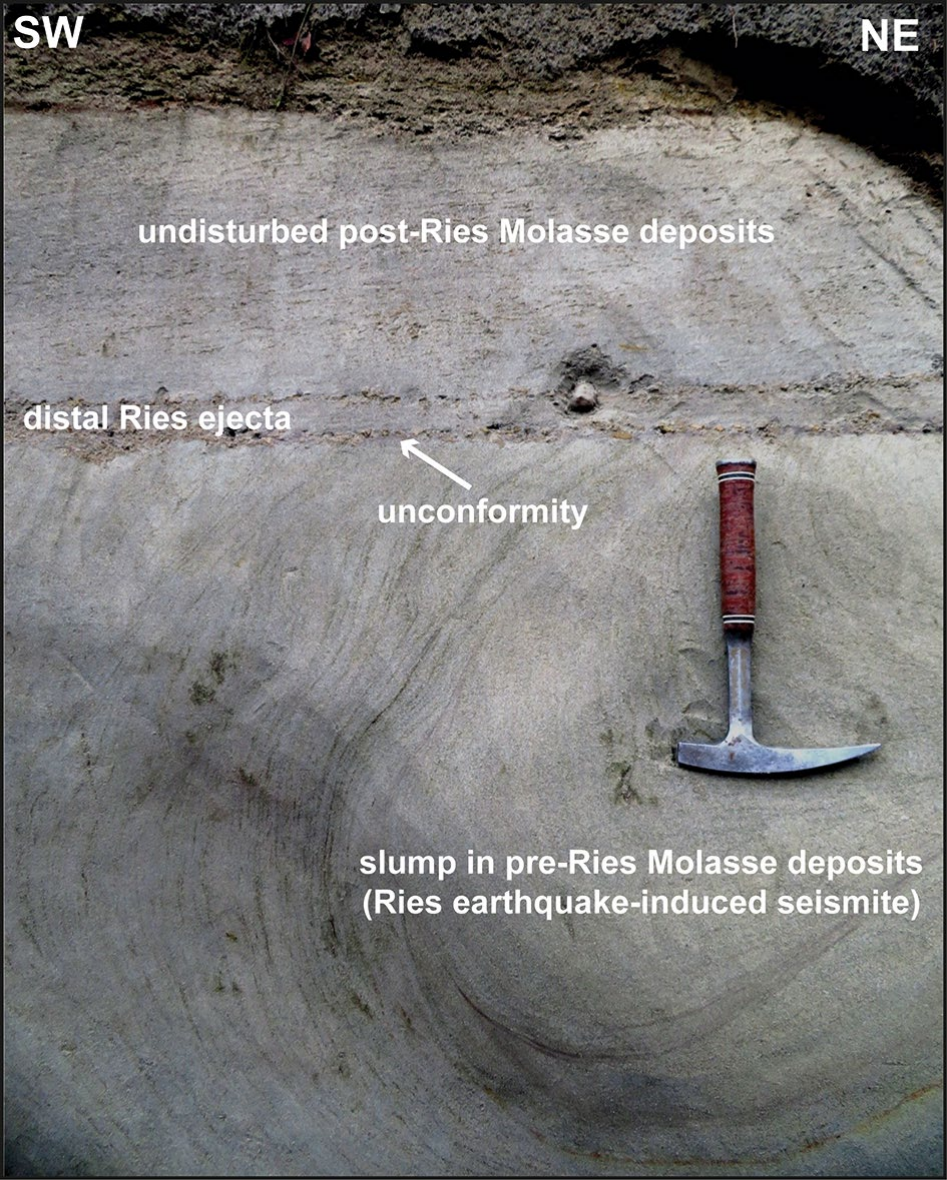
Close-up view of the Kleintobel (Ravensburg) exposure of a seismite unit shown in Fig. 4 exhibiting a distinct slump fold with convolute layering (bottom; hammer in that unit) sharply truncated at its top thereby forming an eye-catching discordance and draped by coarse-grained distal Ries ejecta (pebble right of centre). The rare case of a DREL overlaying a near-perfect unconformity probably reflects a situation where slight elevations of the pre-Ries land surface were cut by the destructive airblast. The DREL shows an internal fining upward trend starting with coarser grained components at its base, overlain by fine sands, and a clayey horizon at its top. It is, in turn, overlain by undisturbed post-Ries deposits characterized by horizontal layering. This exposure of a continental seismite-ejecta couplet highlights the distal environmental effects of the Ries earthquake, ejecta deposition, and the impact-induced air blast in the Mid-Miocene (compare Table 1). Hammer for scale is approximately 30 cm long. Photograph taken in Kleintobel close to Ravensburg by V.J.S.

The Ries impact caused a series of events (Table 1) that affected the wider surroundings of the crater within a minimum radial distance of 180 km^2–4,9–11,15–18,38,49^. Some of the effects overlap and initiated the near-complete destruction of the near-surface environment within this radial distance. The impact-induced earthquake immediately followed the impact event when P-waves reached radial distances of 110 km from the crater centre ~ 15 s after the impact. The earthquake would have lasted for ~ 45 s until P- and S-waves passed this damage zone^57,58^ (Table 1). The seismic energy would have caused intense slumping, soft-sediment deformation, and locally clastic dikes in the upper metres of the water-saturated Upper Freshwater Molasse (Figs. 2, 3, 4, 5, Supplementary Figs. 1 and 2). Approximately 2 min after the impact event, a fire ball and a subsequent air blast^64^ reached the study area blowing off woods, soil, and the upper portions of the slumps and deformed soft-sediments (Table 1). A typical feature of the DREL is that it commonly lies on deformed Upper Freshwater Molasse sediments that are sometimes truncated at the top and exhibit an almost perfectly flat paleosurface (Fig. 4), thereby forming an eye-catching discordance (Fig. 7). This ‘disaster topography’ does not correspond with the original, unaffected palaeolandscape that was dominated by rivers, lakes, and damp forests^5,15,49^. Charred wood, reported for instance from the Unterneul sandpit^18^, suggests high temperatures of the fireball. Within three to five minutes (Table 1), an episode of bombardment by pebbles, cobbles, and boulders mainly of Upper Jurassic limestones, many of them shatter-coned (Figs. 2, 4e), ensued^2,3,10,11,15,16^. The ballistically transported components stem from the uppermost tens of metres of the Ries target rocks^2,10^. They directly overlie the seismite in Upper Freshwater Molasse deposits and sometimes penetrate these sediments by a few cm or dm, thereby forming small funnel-like depressions (Fig. 4). Accordingly, these features can be described as small-scale secondary impact pits (i.e., formed by ejecta projectiles), an impact-related feature rarely seen on Earth^11,15,20^.

**Table 1:** List of the environmental effects of the Ries event from seconds to days after impact affecting the wider surroundings of the impact structure as observed in the study area 100 to 180 km from the centre of the crater.

Event in the study area	Approximate velocity	Environmental effects in study area 100–180 km from impact site	Time and duration after impact event
Earthquake	P-waves: ~ 7 km/s^57^; S-waves: ~ 3 km/s^58^	Seismites: slumps and entire inventory of soft sediment deformation structures, clastic dikes	15–60 s
Fire ball and air blast	Mean velocity ~ 0.5–1 km/s (> 2000 km/h within 5 crater radii for Meteor Crater^64^	Charred wood produced by fire ball^18^; erosion of woods, soils and uppermost seismite-hosting Upper Freshwater Molasse deposits (Fig. 7) by air blast;	130–240 s
Deposition of ballistically transported components of distal ejecta	Starting velocity: 3–4 km/s^10^, fall velocity < 0.2 km/s^10^; mean velocity about 1 km/s; ~ 80 km high trajectory during ballistic transport means ~ 1.7-fold distance compared to linear distance from crater rim to study area	Single cobbles and boulders mainly of Upper Jurassic limestones (some shatter-coned) landed on top of the seismite-hosting deposits, forming a distint discordance (Fig. 7)	170–300 s
Fall-out from impact plume	Mean velocity of vapour plume may exceed escape velocity^2,3^; hot plume velocity of 7–10 km/s^2,3^; collapse starts ~ 2 min. after impact event^2,3^; velocity of ejecta curtain 0.5 km/s 5 km from crater rim^2,3^	Quartz-rich loose sands (sometimes fining-upward succession) forming cm- to dm-thick horizons of distal Ries ejecta from impact plume fallout; sand contains some single shocked quartz grains	Starts ~ 120 s after impact; not earlier than ~ 240 s in the study area, rests for minutes to hours
Heavy rainfall and flash floods	Equivalent to volcanic eruptions, ash from impact plume reaches higher atmosphere and stratosphere by a velocity of some tens of m/s; 70 m/s reported from Mount St. Helens eruption^65^; can last for month if ash reaches stratosphere^66^	Various channels containing reworked distal Ries ejecta incised into seismite-hosting deposits of Upper Freshwater Molasse	Starts minutes to hours after the impact; can last for months

The ejected material temporarily reached a height of ~ 50 to 100 km above the land surface^10^. In contrast to the coarser ejecta fragments, the highly shocked quartz grains were not ballistically transported, but are more likely part of the fallout from the Ries impact plume that began to collapse roughly two minutes after the impact^2,3,10^. Fallout from the impact plume may have rained down for minutes to hours^2,3^. Similar to crustal materials dispersed during volcanic eruptions^65,66^, small airborne ejecta particles and ash from the impact plume probably reached the higher troposphere and stratosphere and caused havy rainfall for days (and possibly for weeks or months due to the atmospheric disturbance) after the impact event.

The Ries impact event was, hence, followed by heavy rainfall and flashfloods (Table 1), as known from volcanic eruptions^65,66^. Fluvial channels were incised into the seismite-bearing Upper Freshwater Molasse in the study area (Fig. 3) and now contain a mix of reworked DREL and locally-derived rock material that can be correlated across several exposures within the North Alpine Foreland Basin. The reworked layers sometimes lack obvious sorting or grading and clasts are matrix-supported. These debritic layers show similarities to lahars to a certain degree. Most of the reworked layers, however, show indistinct sorting, and rounding and imbrication of clasts indicate transport and deposition in fast-flowing, high-energy flood streams (Fig. 3). Logs and pieces of wood up to 2.6 m in length^67^, relics of the impact-blasted wet forest^67^, are abundant in the reworked fluvial deposits. Moreover, well-preserved skeletal remains of the Miocene rhinoceros *Brachypotherium brachypus* were reported in flash flood deposits near Ravensburg^67^. It can be speculated wheter this impressive animal was killed by the hot airblast, struck to death by incoming Ries ejecta boulders, or whether it drowned in the ‘tsunami-like’ continental flashflood following the impact event. In the Biberach and the Ravensburg area, the primary DREL resembles a bone bed owing to the high concentration of fossil wood, remnants of amphibians, reptiles (e.g., turtles, small alligators), and mammals amongst other bones and teeth of rhinoceroses, peccaries, deers (Fig. 4d), water chevrotains, and other hoof animals^67^. The intact nature of bones and teeth document that these fossils were not significantly reworked and that the finding situation is more or less in situ. Some 500 kyr later, the same region was affected by a second set of catastrophic effects, presumably induced by the Steinheim impact event, that produced large dikes cutting through the Ries seismite–ejecta couplet and the overlaying layers of Upper Freshwater Molasse. With the Ries and Steinheim impacts as an extraterrestrial one-two punch, Southern Germany seems to have witnessed a veritable double disaster in the Middle Miocene.

## Methods

### Field studies

In the last three decades, the DREL^10,11,15–18^ was systematically investigated in the North Alpine Foreland Basin. We paid particular attention to ravines in the areas of Biberach and Ravensburg in SW Germany and Bernhardzell (St. Gallen, Switzerland). After heavy rainfall in the Biberach and Ravensburg area in spring 2019, deposits with soft-sediment deformation structures and clastic dikes were partially exposed below and above the distal ejecta horizon along the valley sides of the ravines. The structures were excavated during eight field campaigns from spring to winter 2019. We excavated the sandy foreland basin deposits over a vertical extension of 15 m along the flank of the ‘Tobel Oelhalde-Nord’ (Biberach) and over tens of metres laterally along the flanks of the ravines ‘Tobel Oelhalde-Nord and –Süd’ (Biberach) and Kleintobel (Ravensburg).

### Petrography

Samples of the dike's infills were taken, stabilized by synthetic resin, and processed to polished thin sections. Thin sections of the dike's infill were analyzed for their petrographic properties using a polarization microscope. Additional unconsolidated samples of the infill were investigated by reflected-light microscopy to assess their fossil content (e.g., Miocene mammal bones, invertebrates, and plant remnants).

### Shock metamorphism

Mineral grains separated from the distal Ries ejecta horizon from the Tobel Oelhalde-Nord south of Biberach and Kleintobel near Ravensburg were mounted in epoxy blocks from which thin sections were prepared, then studied using a four-axis universal stage mounted on an optical microscope. Planar deformation feature (PDF) planes in quartz grains and their crystallographic orientation were determined using the universal stage microscope^68,69^. The resulting PDF orientations are indicative of shock pressures that affected the impacted bedrock^68,69^. However, this method requires the detailed analysis of a large number of shocked quartz grains. Due to their rare nature in the distal Ries ejecta horizon, this study does not provide systematic PDF statistics.

### Estimated magnitude of impact earthquakes

Seismic efficiency (i.e., the fraction of the impactor's kinetic energy that is transformed into seismic wave energy) is thought to range between 10^−5^ and 10^−3^. Using a mean value of 10^−4^ for that efficiency^30,56,57^ (and references therein), an equation that correlates the impact energy with the resultant seismic magnitude (M_L_) was derived:

1 $${\text{M}} = 0.67\log _{{10}} {\text{E}}{-} 5.87$$

where M is the local (Richter) magnitude and E is the kinetic energy of the incoming projectile (E = half the projectile mass multiplied with the projectile's velocity squared, in Joules)^30^. Earthquake magnitudes calculated using that equation are only (geologically reasonable) approximations. Applying Eq. (1), the giant Chicxulub impact, for instance, (impact energy ~ 3.7 × 10^23^ J) that caused the mass extinction event at the K-Pg boundary generated a seismic pulse roughly equivalent to a moment magnitude M_W_ 10–11.5 earthquake^20^. The causal relation between the magnitude-distance relation of the formation of seismites in the form of clastic dikes and soft-sediment deformation caused by intense earthquake activity was reported for many regions on Earth^32,37,55,60^. Liquefaction and concomitant formation of seismites caused by meteoritic impact-induced earthquakes is preserved in the sedimentary record^15,17,20,22,23,31,32^ and can help to evaluate intensity of other impact-induced earthquakes. However, the impact earthquake magnitude-distance relationship for liquefaction effects in sediments has to be evaluated mainly from more recent large seismically-induced earthquakes and their distal dewatering effects reported in the literature^59,60^. For earthquake magnitudes given exclusively in local (Richter) scale magnitude M_L_ in the literatue, we estimated M_W_ values (moment magnitude) based on existing M_L_ values. The moment magnitude (M_W_) and local (Richter scale) magnitude (M_L_) are roughly comparable between M_W_ ~ 3.5 and M_W_ ~ 7.0–7.5 for shallow earthquakes (depth < 33 km); at higher magnitudes saturation of M_L_ occurs and the pseudo-linear relationship is no longer valid^70^. M_L_ values for the Ries and Steinheim impacts were calculated using well-established equations ^30^ and impact energy values from the literature^71^. In an additional step, we estimated moment magnitudes M_W_ from reported M_L_ values^30^ by comparing known M_L_ and M_W_ values for historical earthquakes. A range of typical M_L_ and M_W_ values for tectonic earthquakes^59–61,72–74^ and estimates for impact-triggered earthqukes is given in Supplementary Table 1.

## Supplementary Information

Supplementary Information.
