# Supplementary material for: New discovery of two seismite horizons challenges the Ries–Steinheim double-impact theory
Source: Sci Rep. 2020 Dec 17;10:22143. doi: 10.1038/s41598-020-79032-4 (PMC7747748; doi:10.1038/s41598-020-79032-4)
Supplement: Supplementary file 1 — Supplementary Information. [file 41598_2020_79032_MOESM1_ESM.docx]

**New discovery of two seismite horizons challenges the Ries-Steinheim double-impact theory:**

**Supplementary material**

Elmar Buchner*^1,2^ Volker J. Sach^2,3^ & Martin Schmieder^1,2,4^

^1^HNU - Neu-Ulm University of Applied Sciences, Wileystraße 1, D-89231 Neu-Ulm, Germany; E-mail: [elmar.buchner@hs-neu-ulm.de](mailto:elmar.buchner@hs-neu-ulm.de)

^2^Meteorkrater-Museum Steinheim, D-89555 Steinheim am Albuch, Germany

^3^Fokus Natur, In der Talwiese 2, D-72488 Sigmaringen, Germany; E-Mail: [vsach@gmx.de](mailto:vsach@gmx.de)

^4^Lunar and Planetary Institute - USRA, Houston TX 77058, USA; E-Mail: martin@suevite.com

**Description of outcrops**

**Ravine ‘Tobel Oelhalde-Nord‘, Hochgeländ south of Biberach an der Riß
Coordinates: 48º02’24.95’’N, 09º49’52.03’’E**

A large clastic dike is exposed at the flank of this ravine at a topographic height of 598 to 625 meters above sea level (m a.s.l.). The dike strikes along a NNW-SSE direction. The maximum horizontal extent of the exposed portion of the dike is ~17 m. The vertical height of the exposed portion of the dike is 15.2 m (~605–620 m a.s.l.). The length of downward extension and the source layer of the clastic dike are concealed. The thickness of the outcropping part of the Biberach clastic dike decreases from the base (~10 cm thickness) to ~2 cm close to the top. The point of extrusion of the dike at the former land surface was located at 620 m a.s.l.^15^. The clastic dike cuts through a succession of ~15 m of fine-grained, sandy, and commonly cross-bedded sediments of the ‘Fluviatile Untere Serie’ within the Upper Freshwater Molasse^39-41^. At a height of about 2 m above the base of the outcropping part of the dike, this structure cuts through a (at least partially reworked) layer of distal Ries ejecta which demonstrates that the formation of the dike clearly postdates the Ries impact^15^ (for more detailed see description and interpretation in this publication).

The distal Ries ejecta layer (DREL) at ~607 m a.s.l. was excavated over a lateral extent of some tens of meters during field studies in 2019. The DREL is underlain by sandy deposits with a maximum thickness of 1.8 m that exhibit various structures of soft-sediment deformation in the form of convolute bedding, flame structures, and slumps. Fold axes of the meter-scale slumps strike along a WNW-ESE direction. The Upper Freshwater Molasse deposits above the DREL are typically cross-bedded, undisturbed, and unaffected by dewatering processes.

**Ravine‚ ‘Tobel Oelhalde-Süd‘, Hochgeländ south of Biberach an der Riß**

**Coordinates: 48º02’16.08’’N, 09º49’47.06’’E**

At the northwestern flank of the ravine ‘Tobel Oelhalde-Süd’ two horizons of distal Ries ejecta crop out at around 605-606 m a.s.l.. The upper (reworked) horizon exclusively consists of small fragments of Upper Jurassic rocks. Larger limestone components, some of which are shatter-coned, characterize the primary, non-reworked, lower horizon of distal Ries ejecta at 605.3 m a.s.l.^11,15,16^ (see detailed description therein).

**Ravine ‘Wannenwaldtobel‘, Hochgeländ south of Biberach an der Riß
Coordinates: 48º01’50.88’’N, 09º50’01.14’’E**

A primary horizon of distal Ries ejecta crops out at this locality at about 603.5 m a.s.l. and is characterized by angular gravel to cobbles of Upper Jurassic limestones, some with distinct shatter cones in various directions^11^ (see detailed description of the outcrop therein). This horizon is underlain by sandy deposits with a maximum exposed thickness of 2.0 m that exhibit soft-sediment deformation structures in the form of convolute bedding and flame structures. Furthermore, indistinct thrust faults demonstrate the transition of soft-sediment and brittle deformation in weakly consolidated sediments of the Upper Freshwater Molasse. Dip axes of thrust faults strike in a WNW-ESE direction. Molasse sediments above the horizon of distal impact ejecta are, again, typically cross-bedded or horizontally layered, undisturbed, and generally unaffected by dewatering processes.

**‘Liebherr outcrop’ near Ochsenhausen, approximately 10 km east of ‘Hochgeländ’** **Coordinates: 48°04′05.5″N, 9°58′10.6″E**

In the temporary construction site ‘Liebherr outcrop’ (near Ochsenhausen, a suite of at least 2.5 m thickness of sandy sediments of Upper Freshwater Molasse deposits were temporarily exposed around the year 1995^15^. Flame structures and intensely faulted slumps were discovered in these deposits around 600 m a.s.l.. Fold axes of the meter-scale slumps strike in an WNW-ESE direction^15^ (see detailed description of the seismites of this outcrop therein). The stratigraphic level of the Ochsenhausen sediments roughly corresponds to that of the host sediments of the Biberach clastic dike that was recently interpreted to be genetically related to a seismic event induced by the Steinheim impact event^15^. According to the results presented in this study, we associate soft-sediment deformation in the Upper Freshwater Molasse deposits with the seismic event triggered by the Ries impact.

**Ravine ‘Kleintobel‘ close to Ravensburg/Weingarten**

**Coordinates: 47°80'34.20''N, 09°59'85.96''E**

In this ravine, a primary horizon of distal Ries ejecta crops out at a height of ~449 m a.s.l. over tens of meters in horizontal extent. It consists of loose sandy deposits that contain gravels, cobbles, and boulders mainly made of Upper Jurassic limestone and a minor portion of Middle Jurassic limestones and dark claystones and mudstones. The pebbles, cobbles, and boulders are typically angular and sometimes form small crater-like dents in the underlying Upper Freshwater Molasse deposits. Some of the cobbles of Upper Jurassic limestone exhibit distinct shatter cones. The primary distal Ries ejecta layer (DREL) overlays mainly sandy deposits of upper Freshwater Molasse that exhibits conspicuous soft-sediment deformation structures. Convolute bedding, flame structures, and ball-and-pillow-structures are distinctly developed in the deposits and are exposed over ~2.5 m laterally along the ravine flanks. Up to three interfering meter-scale slumps with WNW-ESE directed slumps axes are also exposed. Several dike-structures up to 5 m in vertical extent and about 10 cm in width occur within the convolute-bedded sandy deposits. In analogy to the outcrop ’Tobel Oelhalde-Nord’, a clastic dike (3 mm wide and at least 13.3 m in vertical and 11.4 m in horizontal extent) cuts through the deformed pre-Ries sediments underneath the ejecta layer, the DREL, and the undisturbed post-Ries Upper Freshwater Molasse deposits. In contrast to the dike structure from the ’Tobel Oelhalde-Nord’, the top of this dike is truncated by erosion and capped by Quaternary deposits. The deposits with soft-sediment deformation also show a truncated, erosive top which is overlain by the DREL. The DREL itself is overlain by undisturbed Upper Freshwater Molasse deposits unaffected by water-escape processes.

Horizons of reworked distal Ries ejecta are also exposed along the flanks of the same ravine. These layers exhibit sub-rounded to rounded components of Upper and Middle Jurassic limestones, claystones and mudstones, showing distinct imbrication in places. Several of these horizons erosively truncate each other. The reworked layers of distal Ries ejecta also erosively cut into intensely deformed (slumped) deposits of Upper Freshwater Molasse. The reworked ejecta horizons are overlain by undisturbed, horizontally layered or cross-bedded Upper freshwater Molasse deposits.

**Ravine ‘Bernhardzell an der Sitter‘, St. Gallen, NE Switzerland
Coordinates: 47°28′48.3″N, 9°20′16.9″E**

At this locality, sediments crop out at a height of ~542 m a.s.l. and host the most distal horizon of coarse-grained distal Ries ejecta (‘Ries-Brockhorizont’) currently known^15,17^. Decimeter-long sandstone dikes were reported from these Upper Freshwater Molasse deposits^17^. The sandstone dikes crosscut mottled mudstone that contains distal Ries ejecta^15,17^ (see a more detailed description of the outcrop and detailed discussion of the formation mode of the clastic dikes therein).

**Distribution of seismites**

The Ries and Steinheim impact craters both lie in and are surrounded by Upper Jurassic limestones of the Swabian and Franconian Alb. At the time of the impacts, those limestones had already been diagenetically solidified. Today, all Jurassic and younger sediments north of the Swabian-Franconian Alb escarpment have been eroded. On the Alb plateau south of the craters, Miocene deposits of pre-Ries age (Upper Marine and Upper Freshwater Molasse; or temporally equivalent, correlated sedimentary units) are only preserved in patches. Miocene Molasse deposits that once formed the land surface at the time of the impacts are locally intensely deformed and fractured^18^; however, no seismites have thus far been reported in those deposits. Local deformation (e.g., folding) in the uppermost parts of sedimentary deposits around the Ries crater was ascribed to the outward-directed movement of the impact breccia blanket, as documented in several outcrops. For example, at Ballmertshofen^38^, approximately 15 km south of the Ries crater, a ~5 m-thick unit of marine sands exposed in a sandpit exhibits deformation that is not restricted to the surface, but seems to be pervasive across the entire suite, whereas the underlying Upper Jurassic limestone appears to be undeformed. While such deformation features were previously interpreted as the result of impacting and ground-hugging impact-displaced breccia clods^38^, we now propose that brittle to ductile deformation of these near-surface sediments may have been caused by the major earthquake that immediately followed the Ries impact.

Along the northern rim of the North Alpine Foreland Basin, approximately 30-50 km S and SW of the impact structures, Upper Freshwater Molasse sediments that could potentially correlate with the Ries and Steinheim events are eroded and only deeper parts of the Upper Freshwater and Upper Marine Molasse are exposed. Most exposures of the DREL southeast of the two craters (from the area of Augsburg to the region of Landshut, about 50 to 100 km from the Ries crater) are no longer preserved and are only known from older descriptions in the literature. At a number of sites, the distal Ries ejecta horizon is reworked, locally forming double or multiple apparent layers, and detailed sedimentological descriptions of the underlying bedrock are scarce. Deposits underlying the DREL in the Unterneul sandpit (55 km SE of the Ries crater) are disturbed and clay layers are broken into small fragments, which were interpreted as an effect of impacting limestone cobbles or boulders after ballistic transport^18,38^. Alternatively, those disturbed sediments, too, could be interpreted as a seismite that formed in the wake of the Ries impact event.

**Impact-related earthquakes and related ground-effects**

Most reports on impact-related seismic ground effects stem from sediments at the Cretaceous-Paleogene (K–Pg) boundary where faults at the well-known outcrops near Trinidad, Colorado, and Raton, New Mexico, appear to have been activated following the giant Chicxulub impact event^26^. Likewise, margin collapse and extensive sediment gravity flows triggered by a giant seismic event were reported from the K–Pg boundary in the submarine Gulf of Mexico and Caribbean^24,25,35^. At the K–Pg boundary within the Hell Creek Formation (North Dakota, USA), seismically induced onshore surge deposit overlay sediments with water-escape structures and contain distal Chicxulub ejecta^20^. These sediments with water-escape structures were interpreted as evidence for the accumulation of surge deposits emplaced rapidly out of a dense suspension load^20^. At Moscow Landing, Alabama, USA, in the coastal plain of the Gulf of Mexico, reworked Chicxulub ejecta and tsunami deposits overlie deposits characterized by intense faulting and soft-sediment deformation^22,25,35^. The discovery of (reworked) distal Chicxulub ejecta in context with tsunami deposits and seismites represents a rare case where the effects of impact-related earthquakes and tsunamis are evident in the field^29^. Among the documented examples of palaeo-liquefaction features caused by smaller meteoritic impact-induced earthquakes is a field of circular plugs of sandstone near the Oasis impact crater in Libya, which appear to be the result of upward movement of fluidized sand^25^. Strong evidence for liquefaction and related landslides was also found in shallow submarine sediments in connection with the Chicxulub impact^23-25^. Soft-sediment deformation structures, such as clastic dikes, are widespread in the Paraná basin (Brazil), where they occur at distances between ~50 and ~1000 km from the 40 km-diameter Permo-Triassic Araguainha impact structure^22^. Synsedimentary deformation in the form of convolute bedding and palaeo-liquefaction features, such as iron-oxide cemented sand pipes, were reported from Jurassic rocks in the wider surroundings of the originally ~8 km-diameter Upheaval Dome impact site in southeastern Utah, USA, within a distance of nearly 100 km from the point of impact^31,62^. This suggests that large impact events impart enough energy into the Earth's crust to create extensive volumes characterized by seismites within considerable radial distances from the impact site.

**SUPPLEMENTARY FIGURES**


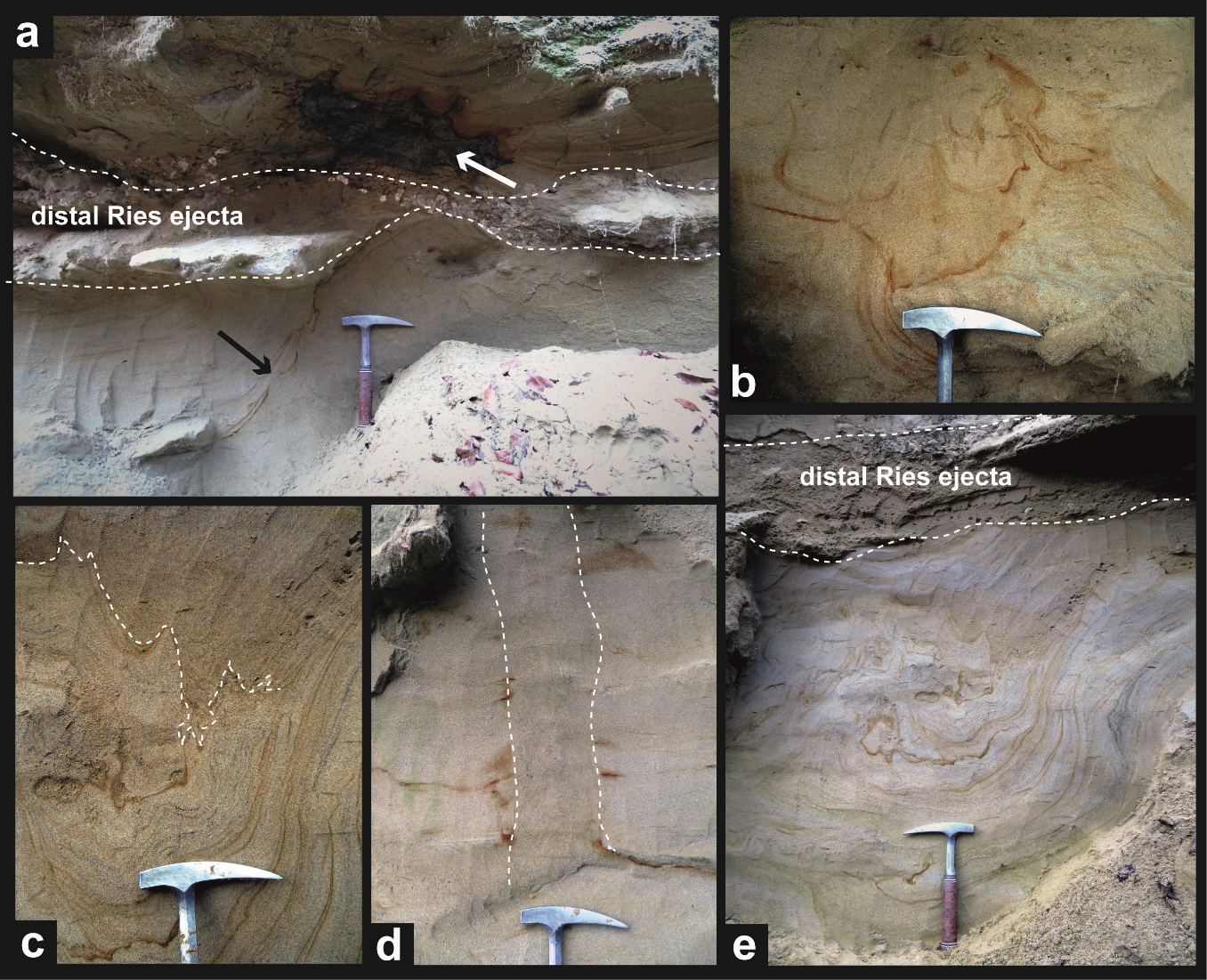


Supplementary Fig. 1: Ries-related seismite and distal Ries ejecta from Biberach an der Riß. a: Undeformed (left) and deformed deposits of Upper Freshwater Molasse with soft-sediment deformation structures subdivided by a zone of brittle to ductile deformation (black arrow) overlain by distal Ries ejecta (Tobel Oelhalde-Nord); white arrow marks remains of wood on top of the distal Ries ejecta layer (DREL). b: Convolute bedding (Wannenwaldtobel) and c: Flame structures (Tobel Oelhalde-Nord); lose-up view of e. d: Dewatering structure in deposits of Upper Freshwater Molasse (Wannenwaldtobel). e: Slump with internal flame structures from Tobel Oelhalde-Nord (hammer head for scale: 20 cm).


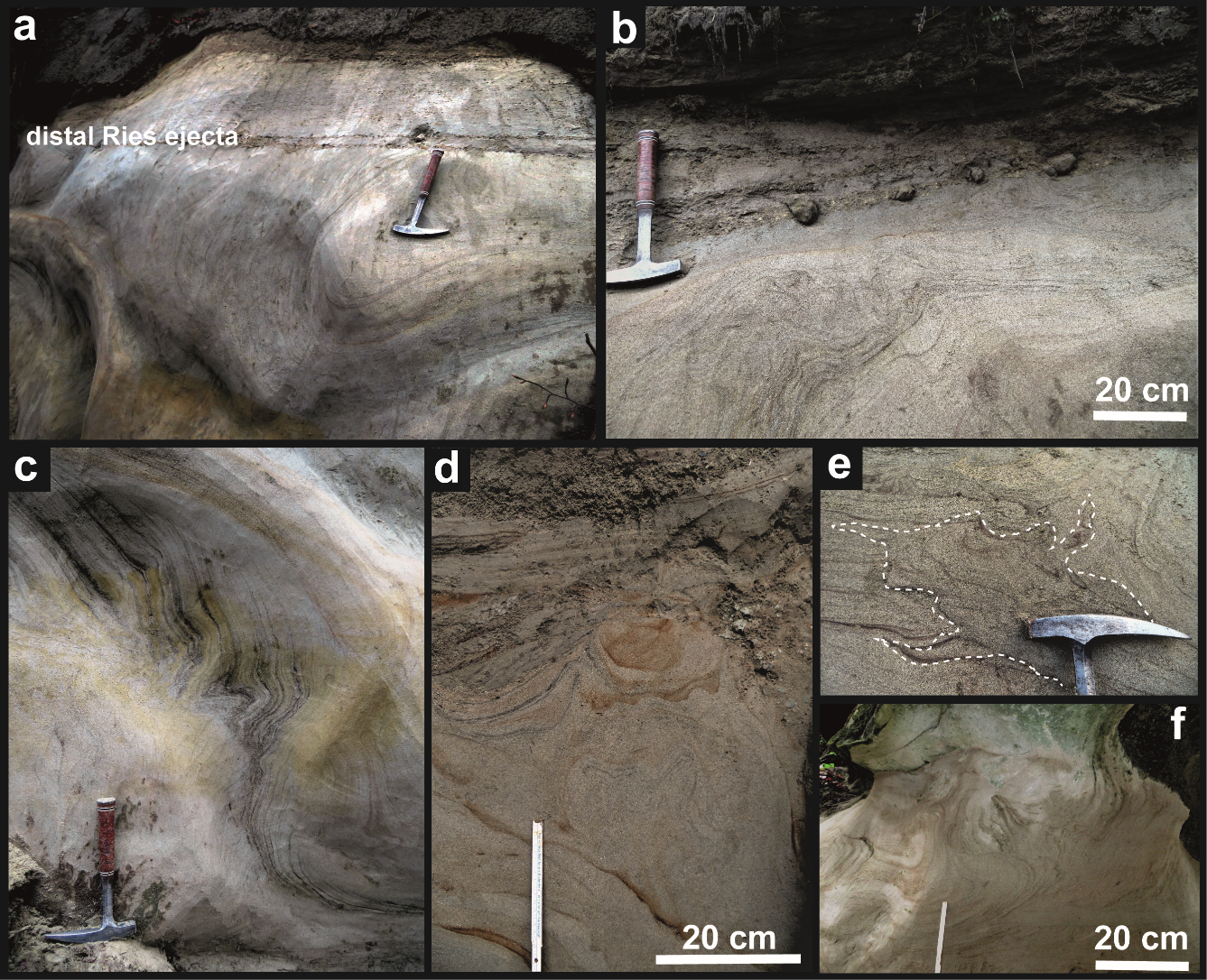


Supplementary Fig. 2: Ries-related seismite horizon and distal Ries ejecta from the Kleintobel near Ravensburg. a: Slump structure (bottom left), and convolute bedding in sandy deposits of Upper Freshwater Molasse. The top of the sediments is flat presumably due to truncation by the impact-air blast and overlain by a horizon of distal Ries ejecta. The Upper Freshwater Molasse deposits above the distal Ries ejecta are completely undisturbed. b: Convolute bedding within Upper Freshwater Molasse deposits overlain by distal Ries ejecta, of which larger pebbles and cobbles penetrate the sandy deposits. c: Convolute bedding within a slump structure. d: Convolute bedding (bottom) and ball-and-pillow structures, and flame structures (e, f) in Upper Freshwater Molasse deposits.


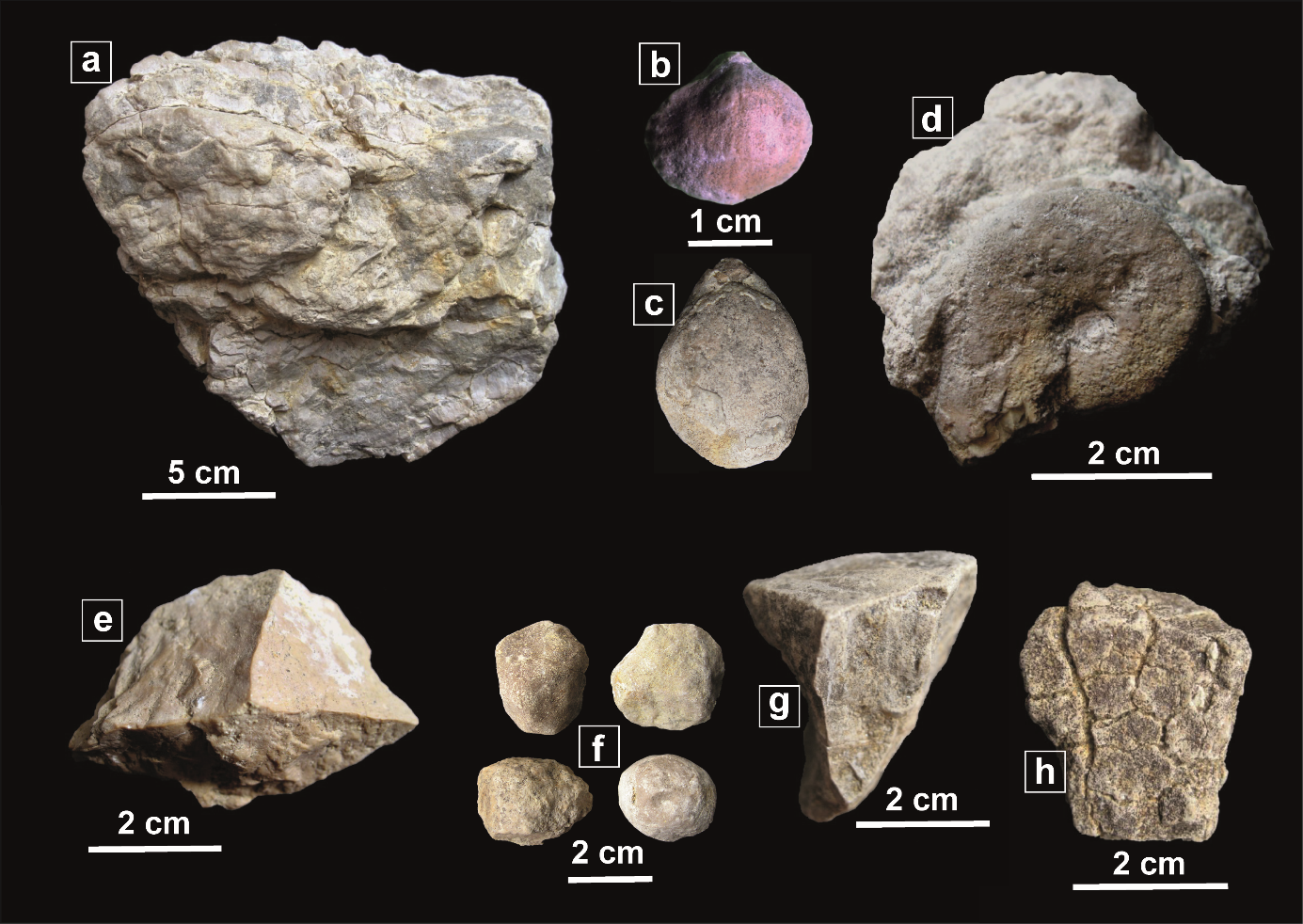


Supplementary Fig. 3: Components of Upper Jurassic limestone in the distal Ries ejecta layer (DREL). a: Intensely fractured, sub-angular cobble from the Wannenwaldtobel (Biberach). b: Rhynchonellid brachiopod from the Kleintobel, Ravensburg. c: Terebratulid brachiopod, Wannenwaldtobel, Biberach. d: Ammonite (*Glochiceras sp.*), Kleintobel, Ravensburg. e: Angular limestone cobble from a primary horizon of distal Ries ejecta, Wannenwaldtobel, Biberach. f: Four intensely rounded coarse gravel components from a secondary (reworked) horizon of distal Ries ejecta, Wannenwaldtobel, Biberach. g: Angular limestone cobble from a primary horizon of distal Ries ejecta, Wannenwaldtobel, Biberach. h: Angular limestone cobble with distinct weathering crust from a primary horizon of distal Ries ejecta, Wannenwaldtobel, Biberach.


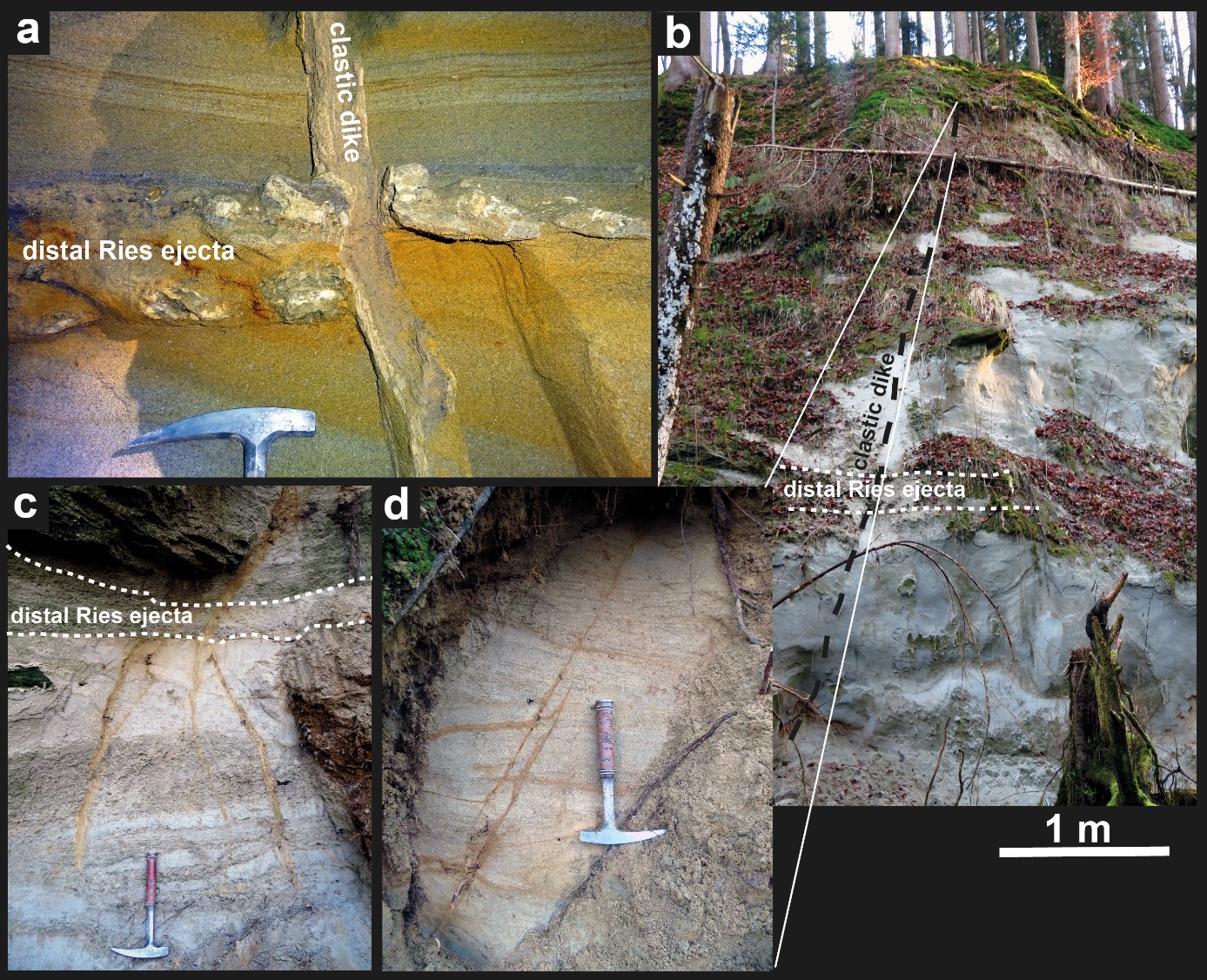


Supplementary Fig. 4: Clastic dikes from the Tobel Oelhalde-Nord near Biberach (a) and the Kleintobel near Ravensburg (b-d). a: The clastic dike cuts through a horizon of distal Ries ejecta. b: Clastic dike cuts through a Ries-related seismite horizon, distal Ries ejecta, and undisturbed overlaying deposits of Upper Freshwater Molasse. c: Clastic dikes cut through Ries-related seismite horizon, distal Ries ejecta, and undisturbed deposits of Upper Freshwater Molasse. The clastic dikes are interpreted as being related to a major palaeo-earthquake presumably triggered by the Steinheim impact. d: Detail of the top of the clastic dike shown in b.


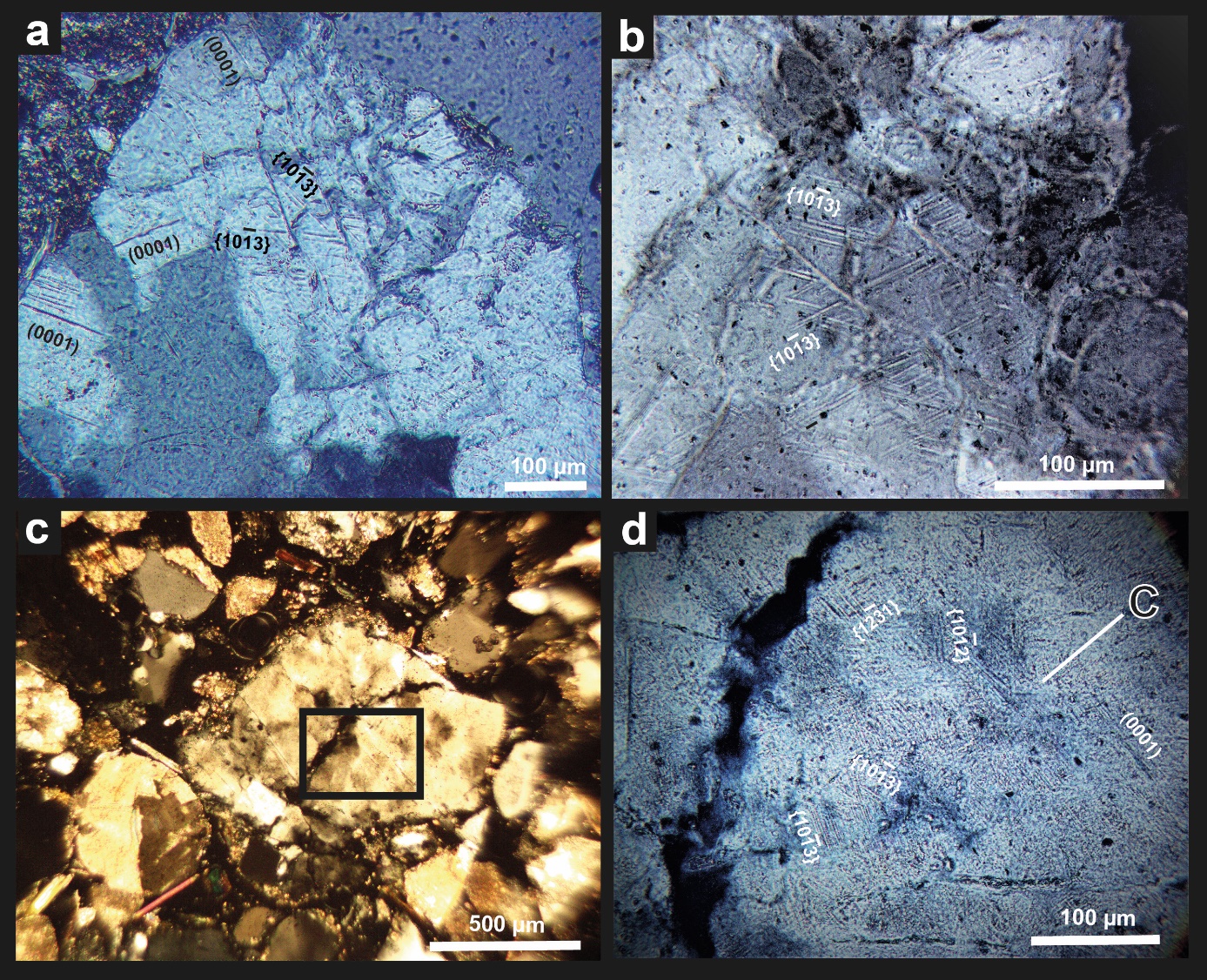


Supplementary Fig. 5: Shocked quartz grains separated from the distal Ries ejecta horizon exposed at different localities. a: Shocked quartz grain with multiple sets of planar fractures (PFs) and planar deformation features (PDFs), Tobel Oelhalde-Nord, Biberach. b: Magnification of a, showing two distinct sets of PDFs. c: Shocked quartz grain with cloudy undulatory extinction, Kleintobel, Ravensburg. d: Magnification of c shows area with six sets of PFs and PDFs, crystallographic orientation indexed for four sets.

Supplementary Table 1: Local Richter magnitude (M_L_) and moment magnitude (M_W_) values for impact-induced and tectonic earthquakes as indicated in the literature. M_W_ for Ries and Steinheim impact-induced earthquakes are estimated from M_L_ values taken from the literature by comparing known M_L_ and M_W_ values for historical tectonic earthquakes.

| Impact-induced earthquakes,  time | Tectonic earthquakes,  year | Local (Richter) magnitude M_L_,  references | Moment magnitude M_W_,  references |
| --- | --- | --- | --- |
| Chicxulub, Mexico, 66.05 Ma |  | ~10^23^* | 10-11.5^20^ |
|  | Valdivia, Chile, 1960 | 8.7^59,60^ | 9.5^71^ |
|  | Alaska, USA, 1964 | 8.7^59,60^ | 9.2^71^ |
| Nördlinger Ries, Germany, 14.81 Ma |  | 8.0^30^* | ~8.5** |
|  | Tokachi-Oki, Japan, 1968 | 7.7^72^ | 8.2^73^ |
| Steinheim, Germany, ~14.4 Ma |  | 6.4^30^* | ~6.6** |
|  | Norcia, Italy, 2016 | 6.1^74^ | 6.5^74^ |
|  | L’Aquila, Italy, 2009 | 5.9^74^ | 6.1^74^ |

*M_L_ values calculated according to^23^ (their eq. 40) using impact energy values taken from^71^.

**Estimated value based on corresponding M_L_ values, which are typically slightly lower than M_W_. The moment magnitude (M_W_) and local (Richter-scale) magnitude (M_L_) are roughly comparable between Mw ~3.5 and Mw ~7.0–7.5 for shallow earthquakes (depth <33 km); at higher magnitudes saturation of M_L_ occurs and the pseudo-linear relationship is no longer valid^70^.
